# Clinical Non-invasive Model to Predict Liver Inflammation in Chronic Hepatitis B With Alanine Aminotransferase ≤ 2 Upper Limit of Normal

**DOI:** 10.3389/fmed.2021.661725

**Published:** 2021-06-02

**Authors:** Shanshan Chen, Haijun Huang

**Affiliations:** ^1^Department of Infectious Disease, Zhejiang Provincial People's Hospital and People's Hospital Affiliated of Hangzhou Medical College, Hangzhou, China; ^2^Graduate School of Clinical Medicine, Bengbu Medical College, Bengbu, China

**Keywords:** liver biopsy, non-invasive model, anti-hepatitis B virus core antibody, liver inflammation, hepatitis B virus

## Abstract

**Background and Aim:** Liver biopsy remains the gold standard for evaluating liver histology. However, it has certain limitations, and many patients refuse it. Non-invasive methods of liver evaluation are thus attracting considerable interest. In this study, we sought predictors of liver inflammation in chronic hepatitis B (CHB) patients with alanine aminotransferase (ALT) levels ≤ 2-fold the upper limit of normal (ULN); these may guide decisions on whether to commence antiviral therapy.

**Methods:** We retrospectively analyzed 720 patients with CHB who underwent liver biopsy and whose ALT levels were ≤2 ULN. The patients were randomly divided into a training and validation set. We used univariate and multivariate regression analyses of data from the training set to construct a model that predicted significant (grade ≥2) liver inflammation, and validated the model employing the validation set.

**Results:** Aspartate aminotransferase (AST) level, prothrombin time (PT), glutamyl transpeptidase (GGT) level, and anti-hepatitis B virus core antibody (anti-HBC) level were independent predictors of significant liver inflammation in CHB patients with ALT levels ≤ 2 ULN. A model featuring these four parameters afforded areas under the ROC curve of 0.767 and 0.714 for the training and validation sets. The model was more predictive than were the individual factors.

**Conclusion:** AST, GGT, anti-HBC, and PT reflect significant liver inflammation among CHB patients with ALT levels ≤ 2 ULN. Their combination indicates whether antiviral therapy is required.

## Introduction

Hepatitis B virus (HBV) infection remains a serious public health problem worldwide. Persistent liver inflammation increases the risk of progression to cirrhosis and hepatocellular carcinoma (HCC) (1). Annually, over 1 billion CHB patients die from chronic liver disease, including cirrhosis and HCC (2). Early appropriate antiviral therapy can inhibit viral replication and prevent disease progression (3).

Treatment decisions for CHB patients are primarily based on the serum levels of alanine aminotransferase (ALT) and hepatitis B virus DNA (HBV-DNA), and the hepatic histological severity grade (grade ≥2 or S ≥2) (4). The AASLD guidelines recommend that patients with ALT levels ≤ 2 ULN, with moderate or severe inflammation, with significant fibrosis, or with elevated HBV-DNA levels should receive antiviral treatment (3). The EASL guidelines recommend that ALT ≥ ULN, moderate or severe inflammation, or significant fibrosis combined with an elevated HBV-DNA level should trigger treatment (1). Liver biopsy remains the gold standard for evaluating liver histology (5). However, the limitations include sampling error, limited dynamic evaluation of the histology, poor patient compliance, and poor intra- and inter-observer consistencies. Most patients are reluctant to undergo liver biopsy (6).

Many researcher seek to replace liver histology with non-invasive diagnostic models, including the aspartate aminotransferase (AST) and platelet (PLT) ratio index (APRI) (7); the FIB-4 test (based on age and ALT, AST, and PLT levels) (8); and the AST:ALT ratio (AAR) (9). These models are commonly used to assess liver fibrosis and cirrhosis. However, few such models have been used to evaluate liver necro-inflammation. There is an urgent need for a non-invasive predictive model based on serum markers that can accurately identify significant (grade ≥2) liver inflammation associated with CHB infection; such a model would facilitate early antiviral treatment decisions.

## Materials and Methods

### Patients

A total of 720 CHB patients with ALT levels ≤ 2 ULN who had undergone liver biopsies were retrospectively enrolled in the Department of Infectious Disease, Zhejiang Provincial People's Hospital, from October 2014 to December 2020. CHB infection was defined as hepatitis B surface antigen (HBsAg)-positivity for at least 6 months (10). The inclusion criteria were confirmed CHB infection and an ALT level ≤ 2 ULN (ULN = 40 U/L). The exclusion criteria were hepatitis C virus (HCV) infection, hepatitis D virus (HDV) infection, co-infection with human immunodeficiency virus (HIV), another cause of chronic liver disease, alcoholic liver disease, autoimmune liver disease, non-alcoholic fatty liver disease (NAFLD), decompensated cirrhosis, HCC, an inadequate liver biopsy sample, and/or incomplete clinical laboratory data. The study was approved by the Ethics Committee of Zhejiang Provincial People's Hospital and all patients provided written informed consent.

### Liver Biopsy

All patients underwent ultrasound-guided percutaneous liver biopsy using an 18 G needle. Biopsy specimens were fixed in formalin, embedded in paraffin, and stained with hematoxylin and eosin (H&E). Each specimen was required to be at least 1.5 cm in length and to contain at least six portal tracts. Histological grading of necro-inflammation (grade 0–4) followed the Scheuer classification system (11). All liver specimens were independently examined by two pathologists blinded to patient characteristics. Depending on the histological changes, patients were divided into a mildly affected group (grades 0–1) and a significantly affected group (grades 2–4).

### Laboratory Tests

Demographic and laboratory data were collected prior to liver biopsy. We recorded age, sex, white blood cell (WBC) count, platelet (PLT) count, prothrombin time (PT), the international standardization ratio (INT), and levels of albumin (ALB), globulin (GLB), alanine aminotransferase (ALT), aspartate aminotransferase (AST), gamma glutamyl transpeptidase (GGT), alkaline phosphatase (ALP), and serum total bilirubin (TBIL). Hepatitis B surface antigen (HBsAg), hepatitis B surface E antigen (HBeAg), and core antibody (anti-HBC) were detected using the CLIA system. The serum load of HBV-DNA was assessed via real-time polymerase chain reaction (ABI 7300 platform, Applied Biosystems, Foster City, CA, USA).

### Statistical Analyses

All statistical analyses were performed using SPSS software ver. 21.0 (SPSS Inc./IBM, Chicago, IL, USA) and GraphPad Prism ver. 8.0.1 software. Continuous quantitative variables were compared using the Student *t*-test or the non-parametric Mann-Whitney test, and are expressed as means ± standard deviations (SDs) or as quartiles. Categorical variables were compared using the chi-square test, and are expressed as numbers or percentages. We used univariate analyses to identify factors significantly associated with necro-inflammatory grade. Then we subjected these variables to multivariate logistic regression to construct a predictive model. The area under the receiver operating characteristic curve (AUROC) was used to evaluate predictive accuracy. A two-tailed *P*-value < 0.05 was considered statistically significant.

## Results

### Demographic Data and Baseline Characteristics

**Figure 1:**
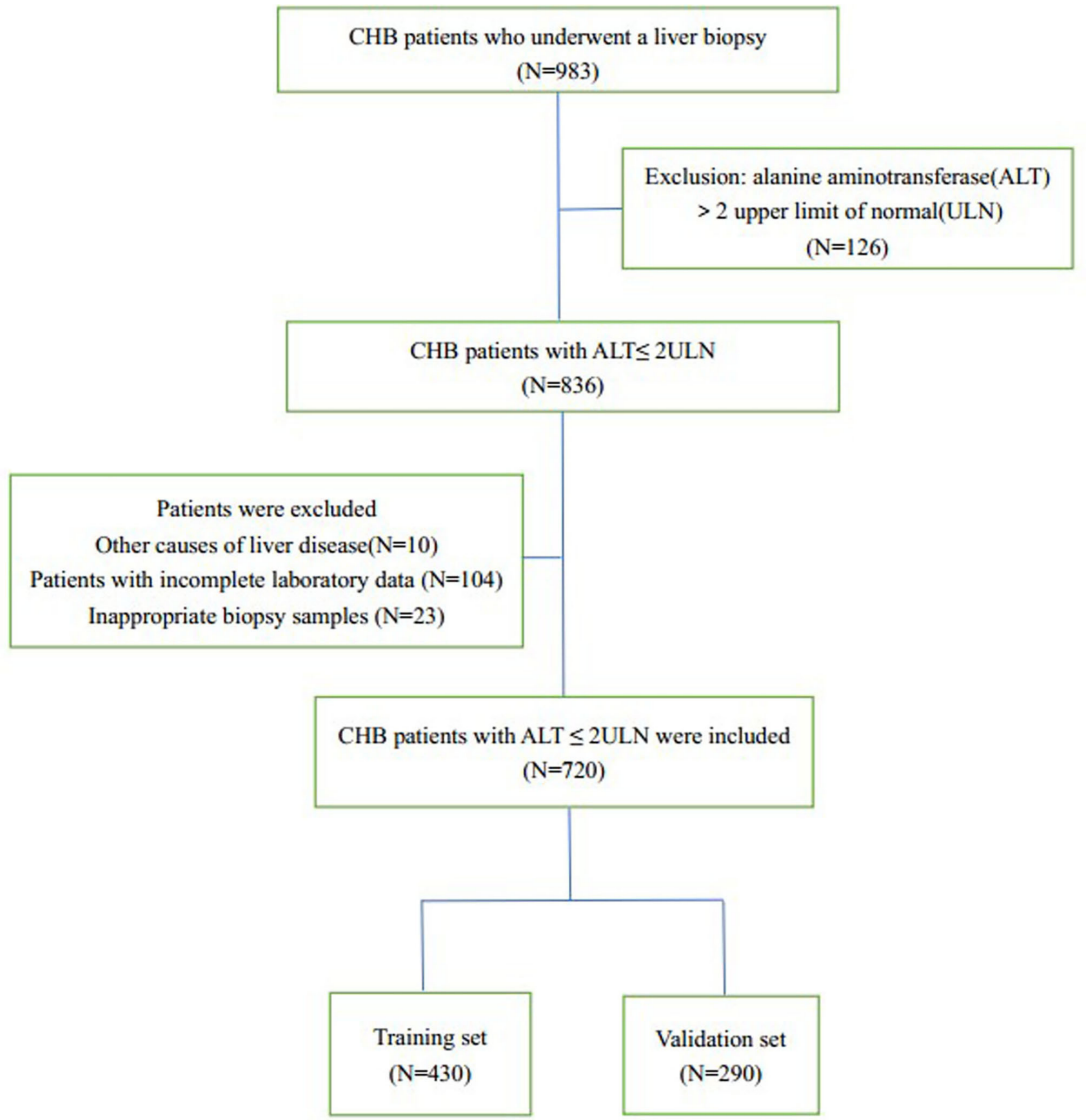
Flow chart of the enrolment of patients. CHB, chronic hepatitis B.

We collected data on 983 CHB patients who underwent liver biopsy in Zhejiang Provincial People's Hospital from October 2014 to December 2020; we excluded 126 with ALT levels > 2 ULN. Of the remaining 826 patients, 10 had other liver diseases, 104 were excluded because of incomplete laboratory data, and 23 were excluded because their liver biopsy specimens were inadequate. Hence, 720 patients were enrolled and randomly divided into a training set (*n* = 430) and a validation set (*n* = 290) (Figure 1).

Table 1 lists all demographic and laboratory data. There were no significant differences between the training and validation sets (all *P* < 0.01). A total of 215 (50.0%) patients in the training set exhibited significant (grade ≥2) liver inflammation as did 138 (47.6%) patients in the validation set.

**Table 1:** Baseline characteristics of patients in the training set and validation set.

**Variable**	**Training set (*n* = 430)**	**Validation set(*n* = 290)**	***P*-value**
Age (year)	38.9 ± 10.1	39.4 ± 10.2	0.418
Male gender (%)	267 (62.1%)	175 (60.3)	<0.001
WBC (×10^9^/L)	5.87 ± 1.60	5.83 ± 1.60	0.896
PLT (×10^9^/L)	190.3 ± 53.9	197.8 ± 125.0	0.941
PT (S)	11.68 ± 0.80	11.64 ± 1.04	0.691
INR	1.01 ± 0.09	1.04 ± 0.62	0.339
ALB (g/L)	44.22 ± 3.46	45.54 ± 21.81	0.926
GLB (g/L)	31.28 ± 18.46	29.55 ± 4.07	0.656
TBIL (umol/L)	16.18 ± 9.36	15.69 ± 6.45	0.720
GGT (U/L)	27.72 ± 22.24	27.12 ± 22.93	0.607
ALP (U/L)	83.86 ± 24.5	86.08 ± 36.8	0.981
ALT (U/L)	33.7 ± 17.6	34.90 ± 17.4	0.291
AST (U/L)	30.0 ± 11.9	30.6 ± 11.7	0.457
HBeAg, positive (%)	193 (44.9%)	121 (41.7%)	<0.001
Anti-HBC (S/CO)	9.58 ± 2.40	13.20 ± 62.07	0.665
Necro-inflammation activity grade			0.602
G0	5 (1.2%)	5 (1.7%)	
G1	210 (48.8%)	147 (50.7%)	
G2	181 (42.1%)	118 (40.7%)	
G3	33 (7.7%)	17 (5.9%)	
G4	1 (0.2%)	3 (1.0%)	
HBVDNA [Log10(IU/ml)]	4.34 ± 2.70	4.46 ± 2.63	0.403

*PT, prothrombin time; INR, international normalized ratio; WBC, white blood cell; PLT, platelet; TBIL, total bilirubin; ALB, albumin; GLB, globulin; GGT, glutamyl transpeptidase; ALP, alkaline phosphatase; ALT, alanine aminotransferase; AST, aspartate aminotransferase; Anti-HBC, anti-hepatitis B virus core antibody; HBeAg, hepatitis B e antigen*.

### Univariate and Multivariate Logistic Regression

Univariate analyses revealed significant differences between mildly and significantly affected patients in terms of PT, WBC count, male sex, PLT count, and levels of ALB, GLB, GGT, HBeAg, AST, ALT, HBV-DNA, and anti-HBC (all *P* < 0.05) (Table 2). Multivariate logistic regression analyses revealed that AST, GGT, anti-HBC, and PT were independently associated with significant liver inflammation (see Table 3 for data).

**Table 2:** Univariate analysis of variables between patients in significant set and no significant set in the training set.

**Variable**	**Significant set (G2-4) (*n* = 215)**	**No significant set (G0-1) (*n* = 215)**	***P*-value**
Age (year)	39.46 ± 10.53	38.3 ± 9.60	0.351
Male gender (%)	131 (60.9%)	136 (63.3%)	<0.001
WBC (×10^9^/L)	5.74 ± 1.60	6.00 ± 1.57	0.044
PLT (×10^9^/L)	184.76 ± 53.36	195.84 ± 54.0	0.050
PT (S)	11.81 ± 0.87	11.55 ± 0.71	0.002
INR	1.02 ± 0.10	1.00 ± 0.08	0.198
ALB (g/L)	43.44 ± 3.80	45.02 ± 2.89	0.000
GLB (g/L)	30.38 ± 4.26	32.17 ± 25.75	0.004
TBIL (umol/L)	16.49 ± 7.54	15.87 ± 10.90	0.238
GGT (U/L)	31.83 ± 25.73	23.61 ± 17.20	0.000
ALP (U/L)	85.83 ± 26.67	81.89 ± 22.06	0.195
ALT (U/L)	39.06 ± 18.06	28.40 ± 15.41	0.000
AST (U/L)	34.57 ± 13.36	25.43 ± 7.82	0.000
HBeAg, positive (%)	114 (53.0)	79 (36.7%)	<0.001
HBVDNA [Log10(IU/ml)]	4.66 ± 2.60	4.02 ± 2.77	0.004
Anti-HBC (S/CO)	10.10 ± 2.12	9.06 ± 2.48	0.000

*PT, prothrombin time; INR, international normalized ratio; WBC, white blood cell; PLT, platelet; TBIL, total bilirubin; ALB, albumin; GLB, globulin; GGT, glutamyl transpeptidase; ALP, alkaline phosphatase; ALT, alanine aminotransferase; AST, aspartate aminotransferase; Anti-HBC, anti-hepatitis B virus core antibody; HBeAg, hepatitis B e antigen*.

**Table 3:** Multivariate logistic regression analysis of independent predictors with significant liver inflammation in the training set.

	**SE**	**OR**	**(95% CI)**	***P*-value**
AST	0.014	1.081	1.051–1.112	<0.001
GGT	0.07	1.014	1.001–1.028	0.042
PT	0.15	1.418	1.057–1.903	0.02
Anti-HBC	0.059	1.320	1.176–1.482	<0.001

*PT, prothrombin time; GGT, glutamyl transpeptidase; AST, aspartate aminotransferase; Anti-HBC, anti-hepatitis B virus core antibody; SE, standard error; OR, odds ratio; CI, confidence interval*.

### Non-invasive Model Predicting Liver Histological Changes

For the training set, the performances of various parameters in terms of predicting significant liver inflammation (grades 2–4) are shown in Table 2. Univariate analyses indicated that PT, WBC, PLT, ALB, GLB, GGT, HBeAg, AST, ALT, HBV-DNA, and anti-HBC differed significantly between mildly affected (grade 0–1) and significantly affected (grade 2–4) patients (all *P* < 0.05) (Table 2). Multivariate logistic regression analyses indicated that PT, AST, GGT, and anti-HBC were independent predictors of disease severity (Table 3). The final model was:

$$\begin{array}{l} 0.078^{*}\text{AST}\left(\text{U}/\text{L}\right)+0.349^{*}\text{PT}\left(\text{S}\right)+0.014^{*}\text{GGT}\left(\text{U}/\text{L}\right)\\ \;+0.278^{*}\text{Anti}-\text{HBC}\left(\text{S}/\text{CO}\right)-3.591 \end{array}$$

### Correlations Between PT, AST, GGT, and Anti-HBC Levels, and Liver Inflammation Grade

**Figure 2:**
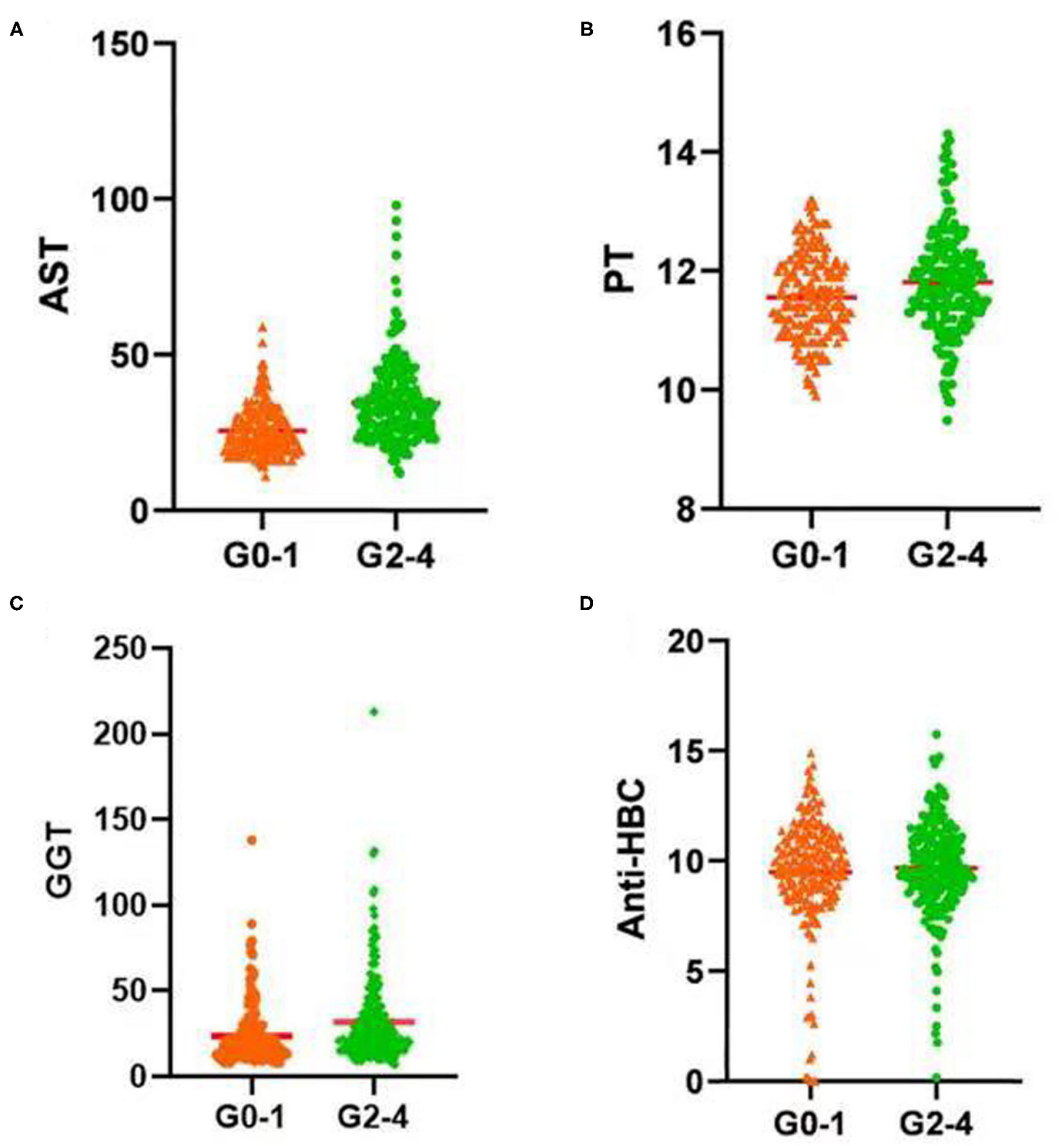
Serum **(A)** AST level, **(B)** PT, **(C)** GGT level, and **(D)** Anti-HBC level increased significantly with increasing inflammation activity grade. AST, aspartate aminotransferase; PT, prothrombin time; GGT, glutamyl transpeptidase; Anti-HBC, anti-hepatitis B virus core antibody; G, inflammation grade.

In the training set, PT, AST, GGT, and anti-HBC differed significantly between the seriously affected group (grade 2–4) and the mildly affected group (grade 0–1) (Figure 2). All of these were significantly lower in the latter patients than in the former patients. Therefore, a model based on these four parameters optimally predicted necro-inflammation levels in the liver.

### Performance of the Four-Factor Combination

**Figure 3:**
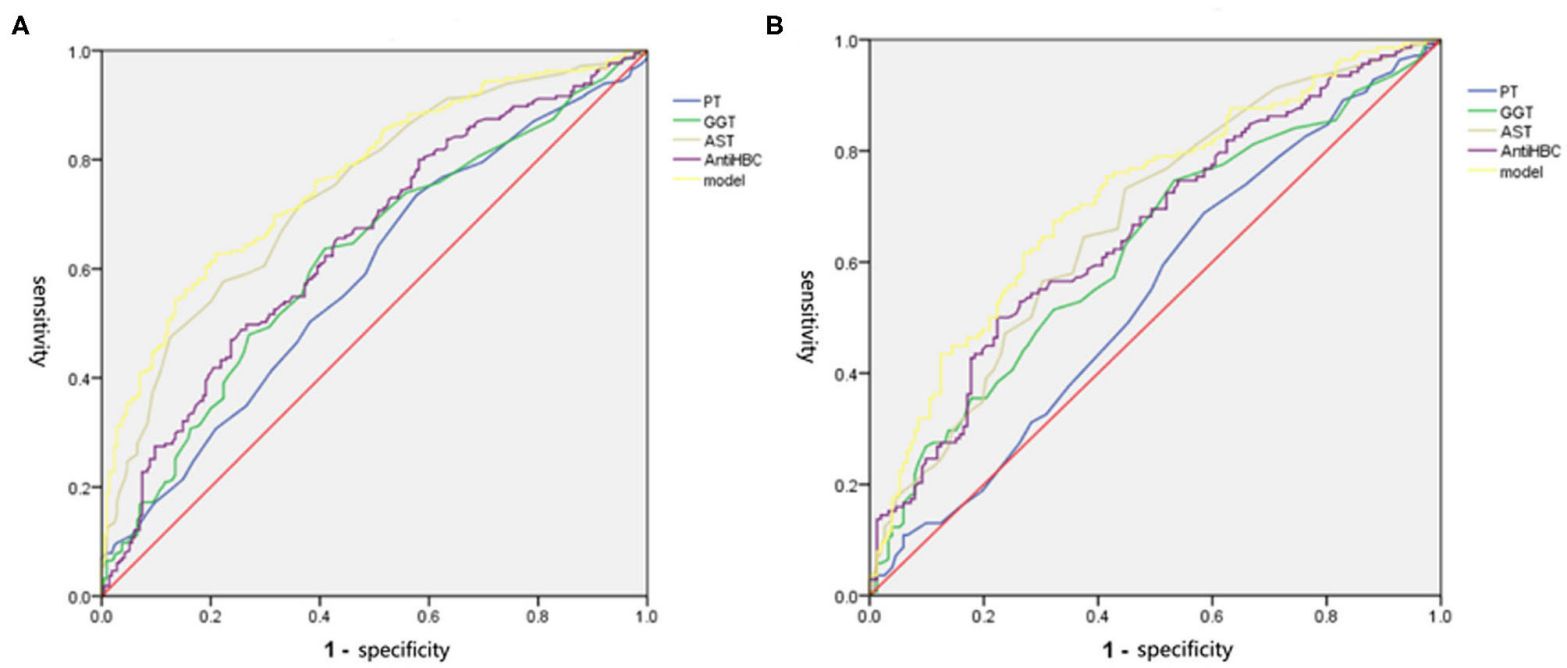
The ROC of independent variables and the model values for identifying moderate to severe inflammation in the training and validation set. **(A)** In the training set, the AUROC of the model values was 0.767, which was higher than that for AST (0.741), PT (0.586), GGT (0.621), and Anti-HBC (0.648) alone. **(B)** In the validation set, the AUROC of the model values was 0.714, which was higher than that for AST (0.674), PT (0.542), GGT (0.624), and Anti-HBC (0.656) alone. AST, aspartate aminotransferase; PT, prothrombin time; GGT, glutamyl transpeptidase; Anti-HBC, anti-hepatitis B virus core antibody; AUROC, area under the receiver operating characteristic curve.

We drew ROC curves. Performance of the noninvasive model for predicting significant liver inflammation in the training set, validation set and total set are shown in Table 4 and Figure 3. The ROC curve of the predictive model for the training set is shown in Figure 3A; the AUROC was 0.767 (standard error [SE] 0.023; 95% CI 0.722–0.811; *P* < 0.001), higher than those for the individual variables. The model sensitivity and specificity for the training set were 66.0 and 76.7%, respectively. The ROC curve for the validation set is shown in Figure 3B; the AUROC was 0.714 (SE 0.030; 95% CI 0.655–0.773; *P* < 0.001), higher than those for the individual variables. The sensitivity and specificity were 67.4 and 67.8%, respectively.

**Table 4:** Performance of the model for identifying moderate to severe inflammation in the training set (*n* = 430), validation set (*n* = 290) and total set (*n* = 720).

	**AUROC**	**95% CI**	**Sensitivity (%)**	**Specificity (%)**	***P*-value**
Training set	0.767	0.722–0.811	66.0	76.7	<0.001
Validation set	0.714	0.655–0.773	67.4	67.8	<0.001
Total set	0.745	0.710–0.781	59.8	77.7	<0.001

*AUROC, area under the receiver operating characteristic; CI, confidence interval*.

### Comparison of the Model With Other Non-invasive Models

We further evaluated the performance of other non-invasive models in terms of predicting significant liver inflammation in the training set. The AUROCs and 95% CIs of the various non-invasive models are listed in Table 5. In general, the diagnostic performances of other non-invasive models were lower than that of our model.

**Table 5:** AUROC of the other non-invasive models for identifying significant liver inflammation.

**Non-invasive models**	**AUROC**	**95%CI**
RPR	0.694	0.589–0.800
H value	0.826	0.786–0.866
ATAR	0.721	0.656–0.780
AAGP	0.77	0.73–0.82
AC index	0.813	0.768–0.852

## Discussion

Accurate assessment of the severity of liver inflammation plays an important role in control of disease progression, dictates subsequent treatment, and predicts prognosis. In recent years, many non-invasive models have been developed for evaluating liver fibrosis and cirrhosis (12, 13). However, few such models have been used to assess the severity of liver inflammation. Therefore, we constructed a non-invasive model to predict the liver inflammation grade of patients with ALT levels ≤ 2 ULN. The model features four independent predictors: AST, GGT, anti-HBC, and PT. The combination of the four parameters was more diagnostic than any individual parameter.

In patients with CHB, persistent liver inflammation is a major risk factor for the development of cirrhosis, HCC, and end-stage liver disease (5, 14). Early and accurate assessment of liver inflammation severity is important. Percutaneous liver biopsy remains the gold standard. However, biopsy is invasive and associated with certain complications (15). Therefore, we built a non-invasive model based on serum markers to predict liver necro-inflammation in CHB patients with ALT levels ≤ 2 ULN.

Currently, the serum ALT level is widely used to assess the severity of liver necro-inflammation (16). However, it is affected by multiple factors (17, 18). Most studies have reported severe liver damage in CHB patients with normal ALT levels (14, 19). In the present study, 215 (50.0%) patients in the training set and 138 (47.6%) in the validation set exhibited significant liver inflammation. An earlier study found that that 64 (37.0%) of 173 patients with ALT levels <64 IU/L evidenced moderate or severe inflammation (20). We consider that the ALT level alone does not adequately reflect the severity of liver inflammation or the appropriate timing of antiviral therapy in CHB patients with ALT levels ≤ 2 ULN. In addition, significant liver inflammation is a major risk factor for CHB progression to cirrhosis and HCC (5, 14).

Some studies have found that the AST level is better for diagnosing liver necro-inflammation than the ALT level (21). A Korean study found that the AST level predicted moderate or severe inflammation with high accuracy (AUROC = 0.78) in CHB patients with ALT levels ≤ 60 IU/L (21). We found that the AST level significantly increased as the liver inflammation grade increased; the level in CHB patients (ALT level ≤ 2 ULN) with significant inflammation was higher than that in patients with mild inflammation. The AST level well-predicted significant liver inflammation; the AUROC was 0.741. Therefore, AST levels should be closely monitored in CHB patients.

Liver injury has many causes. When liver function is impaired, inflammation and cell necrosis activate the coagulation system, and consumption of coagulation-related substances in the liver triggers coagulation dysfunction. The PT reflects the severity of inflammation. The PT was an independent predictor of liver necro-inflammation. As the liver inflammation grade increased, the PT increased; that of the moderate or severe inflammation group was higher than that of the mild inflammation group. The PT reflects hepatocyte synthesis and is associated with a poor prognosis of significant liver inflammation (22). Hepatocyte function and prognosis worsen as the PT increases (22). A PT >5 s that of the control value is prognostic of serious liver disease (23).

The GGT level sensitively reflects the extent of necro-inflammation. It better predicts necro-inflammation than the ALT level, and is an important predictor in patients with HBV infection (2, 24, 25). We found that it independently predicted significant liver inflammation (OR = 1.014, *P* = 0.042). Previous studies have also found that GGT level is a risk factor for significant liver inflammation in patients with HBV infection (24, 26, 27).

The anti-HBC level, a serological marker of HBV infection, can be used to accurately assess moderate or severe inflammation in CHB patients with normal ALT levels (28). One study found that anti-HBC level is independently associated with moderate or severe inflammation in CHB patients with normal ALT levels, with high diagnostic performance (29). Another study showed that anti-HBC level can be used to accurately identify moderate or severe inflammation (AUROCs = 0.768 and 0.767) in CHB patients with ALT levels <64 IU/L (20). We found that the anti-HBC level was a highly accurate independent predictor of liver necro-inflammation (OR 0.824; 95% CI 0.743–0.914; *P* < 0.001). In patients with normal ALT levels, the anti-HBC level increased with increasing liver inflammation, consistent with previous studies (20, 29). However, the mechanism by which anti-HBC antibodies affect liver inflammation during HBV infection remains unclear. Some studies have suggested that the anti-HBC level may affect the responses of B and T lymphocytes (30, 31). The mechanism by which anti-HBC antibody induces hepatocyte injury requires further study.

Our model predicted significant necro-inflammatory activity in the training set; the AUROC was 0.767 (95% CI 0.722–0.811). At a cutoff of 0.56 (the maximum Youden index point) the sensitivity and specificity were 66.0 and 76.7%, respectively. We tested low (0.27) and high (0.64) Youden index cutoffs. The model predicted significant liver inflammation in 183 patients (42.6%) of the training set. Thus, the model was highly accurate, rendering liver biopsy unnecessary for many CHB patients. We also evaluated the performances of other non-invasive models in terms of predicting significant liver inflammation. Most exhibited high diagnostic performances.

An advantage of our model is that it uses routine laboratory data only. A limitation of this study is that it was retrospective in nature. In addition, all 720 patients were enrolled from the same institution. A large multicenter study is required. In addition, our results differ from those of other non-invasive methods; combinations with other methods require study. Finally, the mechanisms of action of the four independent variables (in terms of inducing liver inflammation) remain unclear.

In conclusion, we constructed a non-invasive model based on serological markers. The PT and levels of AST, GGT, and anti-HBC independently predicted significant liver inflammation in CHB patients with ALT levels ≤ 2 ULN. The diagnostic performances were high for both the training and validation sets; the model may reduce the need for liver biopsy.

## Summary

In patients with chronic hepatitis B, persistent liver inflammation is a major risk factor for the development of cirrhosis, hepatocellular carcinoma (HCC) and end-stage liver disease. Therefore, early and accurate assessment the severity of liver inflammation is pretty important for the prognosis of patients. However, clinical application of liver biopsy is limited due to its invasiveness and associated complications. Moreover, most non-invasive models have been developed for the evaluation of liver fibrosis and cirrhosis. Few non-invasive models were used to assess liver necro-inflammation. Therefore, we constructed a non-invasive model based on serum markers for predicting liver necro-inflammation in CHB patients with alanine aminotransferase (ALT) ≤2 upper limit of normal. Aspartate aminotransferase (AST), prothrombin time (PT), glutamyl transpeptidase (GGT) and anti-hepatitis B virus core antibody (Anti-HBC) were related to significant (G ≥2) liver inflammation among CHB patients with ALT ≤2ULN. Combined with AST, PT, GGT and Anti-HBC to construct a model, with area under the ROC curve (AUROC) of 0.767 and 0.714 in training and validation set. The combined indicators have a better diagnostic performance, which may help to reduce the need for clinical liver biopsy, to determine whether to need antiviral therapy.

## Data Availability Statement

The raw data supporting the conclusions of this article will be made available by the authors, without undue reservation.

## Ethics Statement

The studies involving human participants were reviewed and approved by the Ethics Committee of Zhejiang Provincial People's Hospital. The patients/participants provided their written informed consent to participate in this study.

## Author Contributions

SC: collect, analysis data, and perform manuscript drafting. HH: design study and revised the manuscript. All authors contributed to the article and approved the submitted version.

## Conflict of Interest

The authors declare that the research was conducted in the absence of any commercial or financial relationships that could be construed as a potential conflict of interest.

## Abbreviations

CHB: Chronic hepatitis B
HBV: Hepatitis B virus
HBV-DNA: hepatitis B virus DNA
HCC: hepatocellular carcinoma
WBC: white blood cell
PT: prothrombin time
INR: international normalized ratio
PLT: platelet
TBIL: total bilirubin
ALB: albumin
GLB: globulin
GGT: glutamyl transpeptidase
ALP: alkaline phosphatase
ALT: alanine aminotransferase
AST: aspartate aminotransferase
Anti-HBC: anti-hepatitis B virus core antibody
HBsAg: hepatitis B surface antigen
HBeAg: hepatitis B e antigen
ULN: upper limit of normal
AUC: area under the curve
CI: confidence interval
AUROC: area under the receiver operating characteristic.
